# Wind-Induced Fatigue and Asymmetric Damage in a Timber Bridge

**DOI:** 10.3390/s18113867

**Published:** 2018-11-10

**Authors:** Olga Thalla, Stathis C. Stiros

**Affiliations:** Department of Civil Engineering, Patras University, 26500 Patras, Greece; olgath199355@gmail.com

**Keywords:** structural health monitoring, timber, bridge, wind, gusts, lateral deflection, oscillation, fatigue, modal frequency, damage

## Abstract

The transformation of a 30 m long timber pedestrian bridge into a wobbly (laterally swaying) bridge with a dramatically reduced first lateral modal frequency has been monitored by seven annual, multi-sensor surveys. This evidence, in combination with analysis of the wind record, observations of local damage and evidence of wind-induced excitations from other bridges, is used to present a multi-stage scenario of the extraordinary structural weakening of our study bridge in only a few years. Our analysis is constrained by observations of asymmetric damage (longitudinal splitting cracks around metallic connections along the south side of the deck, not explained by ordinary, essentially symmetric lateral oscillations) and over-threshold analysis of strong northerly wind events, including gusts. The proposed scenario is that an unexpected for the area icing event took advantage of construction vicissitudes and produced damage that reduced the lateral stiffness of the bridge, especially of the arch superstructure. In addition, strong winds sharing common direction with gusts produced a combination of semi-static lateral bending and of dynamic oscillations, leading to numerous cycles of asymmetric high amplitude lateral deflections producing tensile stress normal to grain, cracks localized in connections, and fatigue. The vertical stiffness of the bridge was only slightly affected.

## 1. Introduction

**Figure 1:**
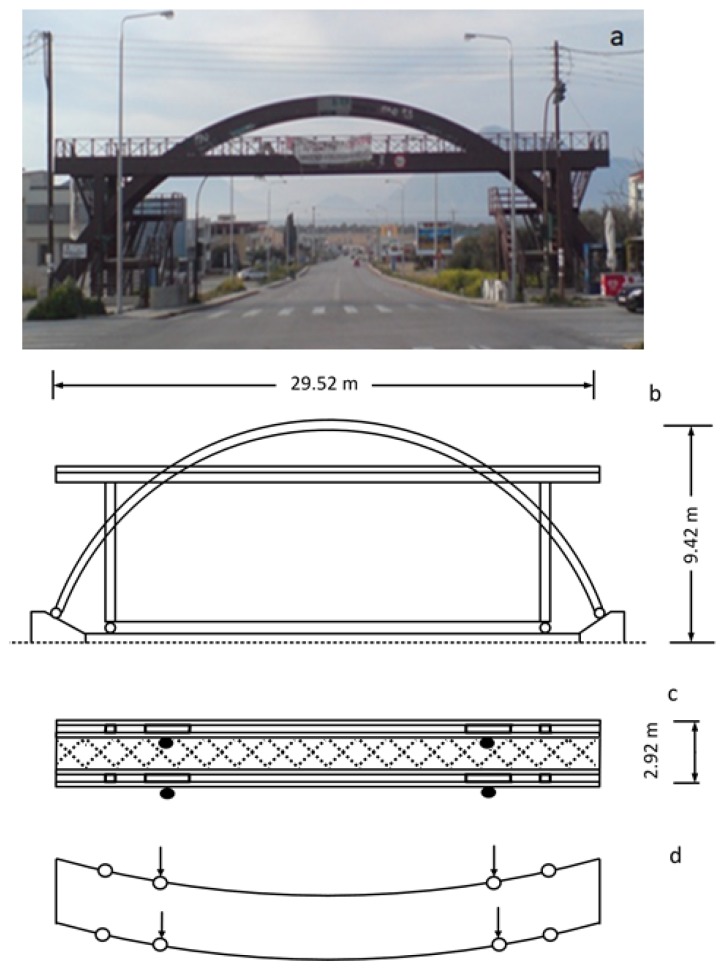
The Kanellopoulos timber bridge and its structural pattern. (**a**) Side view towards north; the sea is visible in the background; (**b**) Simplified vertical structural pattern. High vertical stiffness is inferred; (**c**) Simplified horizontal structural pattern. Low stiffness is readily inferred. Black dots indicate areas of asymmetric damage discussed in Figure 4. X-bracing beneath the deck, omitted in the construction, is marked by dashed x’s; (**d**) Pattern of the instantaneous deformation of the deck because of dynamic loading. Open circles indicate hinges, connections with vertical columns in (**c**) and the arch. Arrows indicate (lateral) hinge loading by the arch structure (see also Figure 5 below).

Analysis of the dynamic deformation of a timber bridge based on systematic, annual multi-sensor records of excitations revealed an extraordinary case of structural decay: after its construction in 2000, the 30 m long Kanellopoulos pedestrian bridge (Figure 1) located at the northern entrance of Patras (Greece), provided a feeling of stiffness and safety to pedestrians. This situation has changed dramatically since 2009, when it was transformed into a swaying, “wobbly bridge” (cf. [1]; see video in the Supplement of [2]).

**Figure 2:**
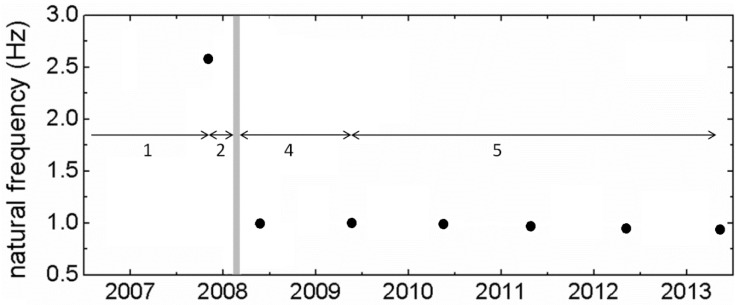
Changes of the first lateral modal frequency and damage stages discussed below. Based on [2,3]. Damage stage intervals are marked with arrows and numbers, stage 3 (icing event) is indicated by a gray line (not in scale).

**Figure 3:**
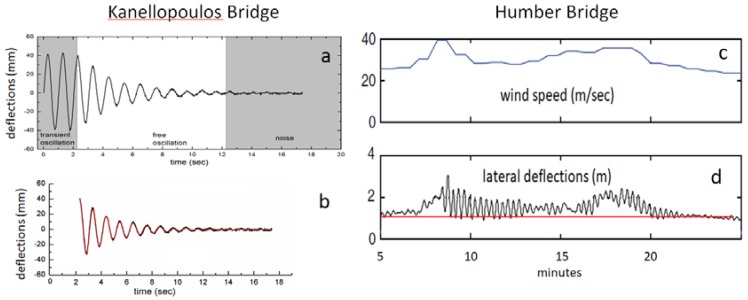
Semi-static and dynamic lateral oscillations in the Kanellopoulos and the Humber Bridge. (**a**) An example of forced, transient oscillation (shaded, left) followed by a free attenuation interval and an equilibrium interval (shaded, right) characterized by noise (after [2]); (**b**) The free attenuation interval in (**a**) (observed, black curve) approximated by an idealized curve (in red). Excellent match of the curves indicates a SDOF oscillation permitting accurate estimation of the first lateral modal frequency; (**c**) Wind record during an extraordinary wind event affecting the Humber Bridge, UK; (**d**) Recorded lateral deflections (deviations from red line) indicate a combination of semi-static bending with amplitude of up to 2 m and of dynamic high frequency oscillations produced by gusts, not shown in (**c**). (**c**,**d**) modified after Figure 17 in [5].

This transformation was recorded by seven annual measurement surveys (2007–2013) focusing on its response to controlled, forced excitations. In particular, analysis of recordings of redundant sensors measuring coordinates, dynamic deflections and accelerations during free attenuating oscillations revealed a change in the first lateral modal frequency of the bridge from ~2.630 ± 0.015 Hz in 2007 to ~1.020 ± 0.001 Hz in 2009. In the following years, an additional gradual reduction of the first lateral modal frequency by 8% was observed (0.990 ± 0.001 Hz in 2010, 0.950 ± 0.002 Hz in 2012, 0.930 ± 0.001 Hz in 2013; Figure 2). These estimates of modal frequencies are reliable and precise because they come from the analysis of free attenuation intervals (nearly perfect, single degree of freedom, SDOF oscillations; Figure 3) of controlled forced oscillations and are confirmed by statistical analysis of acceleration records ([3]; see Appendix A). Because of loss of its lateral stiffness, the footbridge became so flexible and “wobbly” that it was giving the impression of unsafety, if not of an imminent collapse (cf. Eurocode 5 regulations, [4]) and for this reason it was practically abandoned.

**Figure 4:**
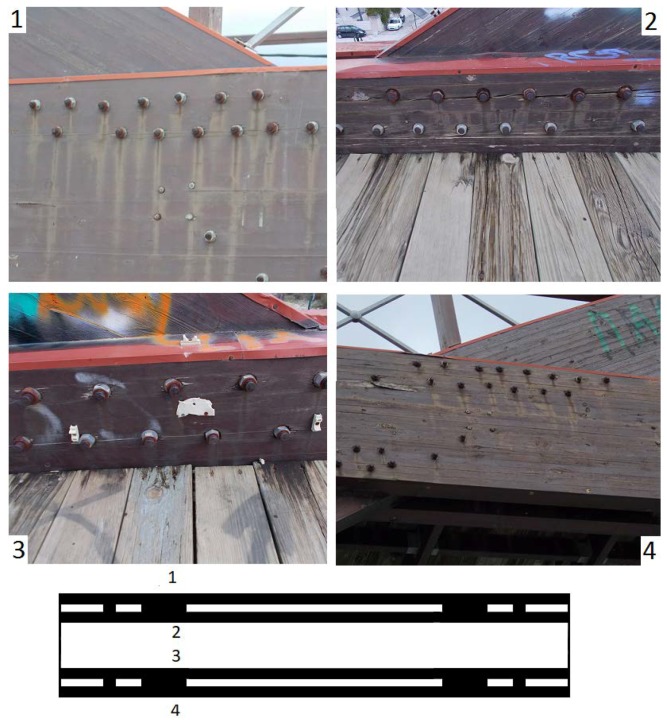
Photos of connections of the bridge deck with the west part of the arch. Location of photos marked 1 to 4 corresponds to connections of the deck with the arch in areas marked with numbers in the deck map at the bottom of the figure (corresponding to Figure 1c). Photos taken in 2012, indicate asymmetric damage (longitudinal splitting cracks), of higher amplitude and frequency along the south sides (2, 4) of the deck beams. A similar situation was also observed in the east side of the deck.

Damage was initially attributed to an unexpected event resulting from a flash icing event in the area (17–19 February 2008, lasting for about 20 h with temperature 0.0 to −1.8 °C), for which the structure, like many other structures in the area, were completely unprepared. Some months later, a magnitude 6.4 earthquake was assumed to have amplified the bridge damage [2]. However, a careful investigation of the bridge revealed asymmetric structural damage, longitudinal splitting cracks around key hinges connecting structural members, mostly on the south faces of the bridge deck (Figure 1d and Figure 4). Such observed *asymmetric* damage cannot be directly assigned to ordinary excitations which produce nearly *symmetric* oscillations (Figure 3).

This result led us to investigate the structural decay of this timber bridge, searching for processes which can produce *asymmetric* damage. In this framework, all available data were re-examined: the bridge construction pattern, its defects and damage, data collected and analyzed during our experiments and the inferred changes of modal frequencies, the available photos and videos archives, metadata (especially notes on loading conditions and feeling of the parties contributing in the experiments), information on the possible loading types and on the response of the bridge to various types of loading and to environmental effects, as well as information on the feeling of the pedestrians crossing the bridge.

The output of this study is that damage in the bridge was produced in an unusually short period (a few years) by the combination of two effects: of an extraordinary for the area icing event which took advantage of structural vicissitudes and damaged the bridge reducing its stiffness, and then by fatigue, corresponding to numerous cycles of asymmetric lateral wind loading on the deck, amplified by the movement of the superstructure (arch). This was possible because fatigue appears in wood after fewer cycles of strain than in other structures, and wood is vulnerable to tensile stress normal to the grain especially at connections. Loss of stiffness of the arch superstructure because of damage, permitted larger amplitude lateral oscillations which amplified this stress.

The particularity of this study is that it is not simply based on analysis of experimental data to detect damage [6], but it combines analysis of monitoring and of wind data with observations of damage and with evidence from other bridges to explain the sequence of events which led to a laterally swaying bridge.

## 2. Structural Pattern

The Kanellopoulos timber footbridge (Figure 1) was constructed in 2000 using GLULAM wood and certain steel elements in an essentially green field area with low-lying (up to two story buildings) at the northern edge of Patras. The deck is oriented in the NE direction (N 43° E), has a total length of 29.5 m and width of 2.91 m and is formed by two main twin horizontal parallel beams, each with 110 × 504 mm cross section, stiffly connected at their edges by two transversal beams. The deck is supported by four vertical columns (cross section 110 × 504 mm) defining a midspan of 26.5 m and by two vertical arches (cross section 110 × 630 mm) which are sandwiched between the two main twin horizontal beams (Figure 1c).The connections of columns with the horizontal beams are made with two sets of bolt hinges with screw nuts without washers (28 bolts in total for each arch column, 16 for each edge column; Figure 4).

**Figure 5:**
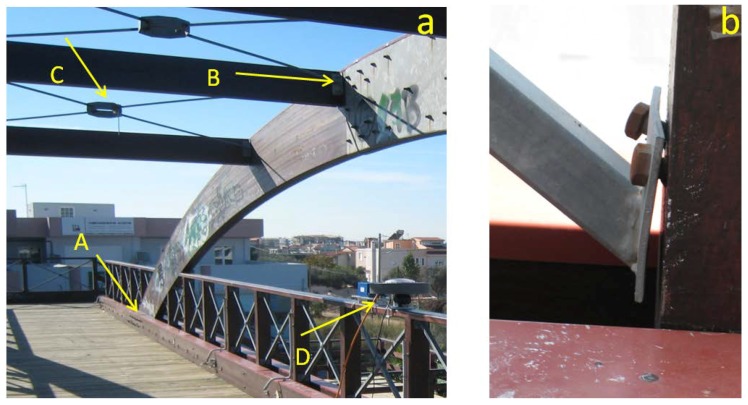
(**a**) Photo of the bridge during the 2007 survey, before damage was observed. Arrow (A) points to connections (2) in Figure 4, in which no signs of splitting are visible. Arrow (B) points to transversal roof beams, which appeared strongly coupled with the two arches. Arrow (C) points to metallic elements, unable to offer rigidity and which were sheared after 2009. Arrow (D) points to some of the collocated sensors (accelerometer, GPS, RTS reflector) used during the annual monitoring surveys; (**b**) A characteristic example of ice-extruded bolt; from a photo taken in 2009.

The deck floor is formed by timber slats supported by transversal beams with spacing of about 1m, and between them two lines of beams, parallel to the deck edges. X-brace stiffeners below the deck were included in the initial plan (Figure 1c), but they were omitted in the construction, and this proved critical for the bridge health, as explained below. The two vertical arches were connected at their top by sub-vertical transversal beams and metallic X-type braces (Figure 5). The latter, however, were not rigid enough to play the role of stiffeners. For this reason, the arch superstructure tended to sway.

Hence the bridge can be regarded in a first approximation as a horizontal truss frame supported near its four edges by four vertical columns and by four columns produced by the two arches (Figure 1). This leads to an initial structure very stiff in the vertical axis (main vertical modal frequency of the order of 6.5 Hz [7]) and flexible along the lateral axis (main lateral modal frequency of the order of 2.6 Hz), but satisfying Eurocode regulations (cf. [4]).

## 3. Non-Instrumental Evidence of Structural Damage

Till 2007 the bridge was giving the impression of stiffness and security, and its lateral deflections were small, and this was reflected in the results of our 2007 survey, indicating a first lateral modal frequency of 2.6 Hz [2]. Since 2009 the bridge was transformed into a wobbly structure and its swaying was evident when crossed by groups of people or even when excited by wind (see video in Supplement in [2]). Extreme deflections were accompanied by noise (creaking), testifying to progressing small scale damage. Significant wobbling, however, occurred only if excitations were made by groups of people (approximately, total mass >500 kg) or winds with minimum intensity approximately 6 in the Beaufort scale (~50 km/h, 14 m/s, empirically derived from the roughness of the sea surface visible at a distance of ~750 m from the bridge; see Figure 1a).

Analysis of photos and videos and of various data reveal that the structure was free of damage in 2007, while those in 2009 and later indicated signs of important structural damage (Figure 5; [2]); this damage was especially clear around metal connections (Figure 5b). A careful visual investigation in 2012 revealed that the structural damage in the bridge was essentially of three types: (i) extrusion and partial failure of certain metallic connections, (ii) sheared (plastically deformed) stiffeners on the top (arch superstructure Figure 1 and Figure 5) and (iii) longitudinal splitting cracks in all four connections between columns and twin beams, but mostly along the southern faces of the two twin beams (Figure 5). The first two types of damage have been discussed in [2] and provide no evidence of asymmetry in damage distribution; the third type of structural damage, on the contrary, is asymmetric and systematic, and is the focus of this article.

Examination of photos and videos revealed that significant sub-horizontal longitudinal splitting cracks have been observed since 2009 in the two twin horizontal beams supporting the deck around the four sets of hinges clumping beams with the two curving columns. The amount and the dimensions of such cracks in the two sets of horizontal beams were systematically more frequent and much larger on the southern faces of the bridge than on the northern ones (Figure 4).

Around 2012 the length of these cracks ranged between a few and about twenty centimeters long around each individual bolt, but more recently (2017), they have been amplified in number, length and width, and their pattern has become more chaotic. These longitudinal splitting cracks clearly did not exist during the first survey in 2007 (Figure 5a).

Environmental effects, for example increased exposure of the southern sides of the bridge to rain and sun radiation leading to loss of wood humidity could have led to an asymmetric decay, but with a rather uniform distribution along the whole southern faces of the beams. Typically cracks like those of Figure 4 are a result of stress, and they tend to propagate along the grain direction as a consequence of the low resistance of wood to tension perpendicular to the wood grain [8], because of secondary, and not of primary extension (cf. [9]). Damage was possibly amplified due to the lack of washers on screw caps [10].

## 4. Instrumental Study of the Bridge Decay

Between 2007 and 2013 the dynamic performance of the Kanellopoulos bridge was evaluated on the basis of the analysis of instrumental data in combination with visual inspections [2], photos and videos and reports for the feelings of pedestrians crossing the bridge. These surveys were made once per year (in November 2007 and in May during the following years) lasted for a few hours (so that environmental conditions were unchanged during a survey) and were based on analysis of the free excitation intervals of controlled, forced oscillations (SDOF; Figure 3a,b) under essentially uniform environmental and loading conditions based on redundant sensors, mostly collocated (Figure 5). Three types of sensors were used: accelerometers, geodetic sensors measuring dynamically changing coordinates of selected points on the bridge, GPS and robotic theodolites (RTS; Total Positioning System sensu [5]), and a distance meter (Tellurometer) measuring length changes of a selected point on the bridge from a station on stable ground. Video-recordings of the response of the bridge to the excitations (such as those in Supplement of [2]) were also analyzed. Different types of well-controlled forced controlled excitations (cf. [2,11,12] were made in each survey, collected data were carefully processed, and intervals characterized by free attenuations of oscillations were identified (for details [2]. More details are given in Appendix A.

Obtained sets of measurements in each survey are free of the bias imposed by environmental and operations conditions (temperature, etc. [13,14,15]) and from the statistical point of view they represent stationary data sets. From the analysis of essentially SDOF oscillations of free attenuation intervals during each survey (Figure 3), the dominant frequency of each excitation event was computed with precision. Approximation of attenuation intervals by typical equations (e.g., [16], Appendix A.3) confirm that the analysis is based on SDOF free attenuating oscillations describing the first modal frequency. Consistent results from different quasi-similar excitations and sensors were used to derive the first lateral modal frequency of the bridge for each survey, summarized in Figure 2 (see details in Appendix A). This figure is representative of the true changes of the dynamics of the study footbridge, because instrumentation, environmental conditions, loading and analysis techniques were essentially uniform in all surveys and results were consistent with the feelings of the party producing the forced excitations. Furthermore, a characteristic of forced excitations is that they produce nearly *symmetric* lateral oscillations (Figure 3a). Hence such loading cannot easily explain the observed *asymmetric* damage of Figure 4.

Apart from deflections of the deck, lateral deflections of the top of the arch superstructure were also analyzed during certain surveys after 2009. The outcome of these analyses is that during free attenuating oscillations the amplitude of the oscillation of the top of the superstructure was 7% higher than that of the deck, but 43% higher during steady/transient oscillations (64.3 to 60.2 mm and 90.2 to 64.0 mm, respectively, between two consecutive high and low peaks, 2013 survey data). This implies increased strain on the bridge during steady/transient oscillations (Figure 1d).

Another characteristic is that lateral deflections recorded at the mid-span of the deck were of the order of 2 cm in the first campaign (2007) but increased to up to 8–10 cm in the 2013 and most recent surveys under comparable loading and environmental conditions (see video Supplement in [2]) showing a case of oscillation with increased amplitude). Such deflections are extraordinary, and their amplitude can be understood in comparison with those of an about 130 m long, cable-stayed timber bridge for which measurements are available: lateral deflections at the mid-span of this last bridge were of the order of 1 cm during excitation by strong winds (wind speed 14.5 m/s, about 50 km/h, [17]).

## 5. Possible Causes of Asymmetric Damage

We examined all possible loading-associated scenarios which may explain the observed asymmetric damage confined to connections:

(1) decay because of loading during normal or abnormal use by pedestrians, especially forced excitations by youngsters
(2) earthquake-induced oscillations
(3) loads from atmospheric effects, such as temperature effects, wind-induced effects, either usual or extraordinary, including vortex-induced vibrations, and combination of wind and rain and of wind and ice
(4) loads associated with extraordinary events such as impact by vehicles, etc.
(5) uneven loading of the deck due to deformation or weakening of foundations or of its columns, including biological etc. decay
(6) material inhomogeneity leading to asymmetric damage
(7) decay of wood because of loss of humidity

Among these scenarios, #4–7 can be readily discarded for lack of the necessary evidence. Scenarios #1 and 2 can also be discarded, because they produce only nearly symmetric deflections and damage (see Figure 3a). The remaining possibility is hence #3.

The effect of rain associated with wind has been studied mostly in the cables of long bridges [18], but in a short bridge it could have played a minor role only. Ice plays an important role various structures because it tends to reduce their modal frequencies [19], but in the study bridge, which is in a location in which temperatures are always above 2–3 °C (and hence various structures are unprotected to ice effects), the role of an icing event may be critical.

Wind in generally excites bridges producing oscillations along its direction, or normal to it (aeroelastic effects), but wind effects are usually confined to long, mostly suspension bridges (cf. [18,20,21]). In addition, no clear correlation documenting a causative relationship between wind and small-scale variations of lateral displacements of bridges exists (e.g., Figure 24 in [22]).

Recently, however, it was realized that strong winds produce a combination of semi-static and of dynamic deflections (Figure 3d; [5]). The impact of wind on smaller, timber bridges remains unknown, and only recently there has been presented evidence of wind-induced lateral deflections of a pedestrian bridge [17]. Furthermore, decay of the southern faces of the deck because loss of humidity due to uneven exposure to sun radiation and an overall material deterioration cannot be excluded, but it is unlikely to represent a dominant cause for observed damage only near connections.

## 6. Wind Impact on the Bridge

### 6.1. Available Data

We analyzed wind data which cover the period for which instrumental data describing the bridge decay are available, between November 2007 and May 2013. Data come from a station initially ~400 m SW of the bridge. In 2010 this station was shifted at a location ~800 m SW of the bridge. In both locations, the meteorological station was located at the top of two-story buildings, in environments unobstructed by other buildings. The wider study area is flat, adjacent to the sea and partly covered by low-rise (typically less than 8 m high) buildings spread in green fields. For this reason, the available wind speed measurements for both stations are fully representative of the wind conditions at the bridge.

Wind characteristics were measured with a WIRELESS VANTAGE Pro2 weather station, produced by Davies Instruments (www.davisinstruments.com). Wind speed and direction were among the atmospheric parameters (temperature etc.) measured approximately every minute but down-sampled and stored to successive 5-minute intervals *t_i_*. The wind data analyzed are in the form of CSV files corresponding to sets of three scalar variables:

*v_i_* = (*t_i_*, *W_i_*, *θ_i_*), *i* = 1, 2, … *n* (1)

describing the maximum wind speed *W_i_* and its azimuth *θ_i_* in time intervals *t_i_* covering the whole study period with no significant voids.

### 6.2. Analysis of Wind Data

**Figure 6:**
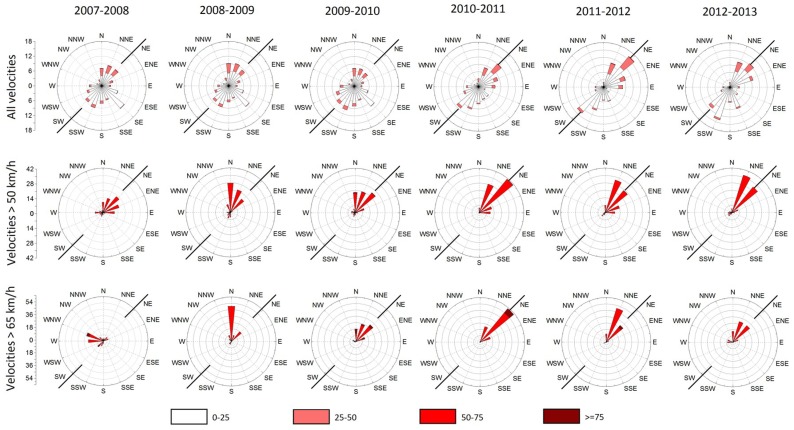
Rose diagrams of the wind distribution during the six intervals defined by the bridge measurement surveys. Scale indicates percentage of occurrence. Two black lines in NE-SW direction indicate the main bridge axis. Top: all winds; middle: winds >50 km/h; bottom: winds >65 km/h. Mark that strong northerly winds with a significant component normal to the bridge axis are confined to the interval 2007–2008, when most of the damage occurred.

The distribution of wind characteristics in the intervals defined by our six annual structural health monitoring surveys is summarized in the form of rose diagrams in the top line of Figure 6.

**Figure 7:**
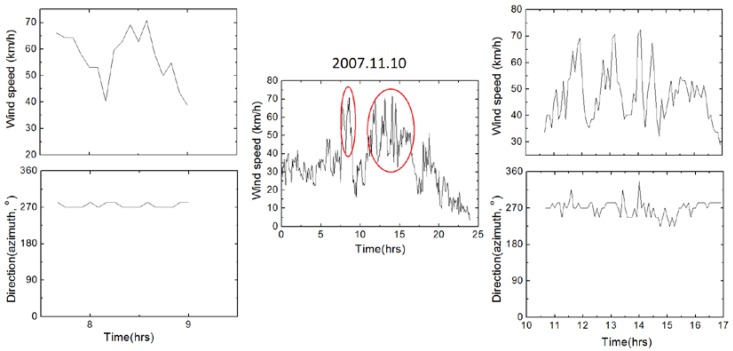
Details of a representative strong wind event (2007.11.10), consisting of two strong sub-events (marked by red ellipses). Details of velocity and azimuth changes are shown in the two edge columns. During the two sub-events one and five hours long, recorded wind was nearly stable in direction, ranging between approximately 30 and 70 km/h. Graph based on max winds in successive 5 min long intervals.

The analysis of the available wind record permits to recognize that the broader area is characterized by long intervals of no or of very low winds, interrupted by intervals of strong winds (typically above 40 km/h), occasionally lasting for up to three days. The intervals of strong winds are thereafter regarded as wind events, and sometimes included two or more sub-events. All these wind events were separately analyzed, and a representative example is shown in Figure 7. This analysis revealed that during a wind event the wind direction remained relatively stable (azimuthal variations of usually less than 10–20°), and the wind speed was also kept essentially above a certain threshold (Figure 7).

The analyzed data, however, derive from low-frequency records, and do not cover gusts. Evidence, however from studies in nearby structures, including high rate instrumental recording of wind and video recordings of its impact various in various structures etc. indicate that gusts in the area systematically accompany strong winds, and flow from the same direction.

Because of their stiffness, bridges are excited by winds only above a certain threshold and when flowing from specific directions [23]. This result is consistent with empirical evidence from pedestrians systematically crossing the Kanellopoulos bridge, and who were feeling its lateral excitation only by strong northerly winds, accompanied by sounds of creaking; from observations of roughness of the nearby sea this threshold corresponds to northerly winds of intensity 6 in the Beaufort scale, approximately 39–49 km/h, or 11–14 m/s.

For this reason, we focused on the strong winds which occurred in this area and using the available data we compiled rose diagrams for significant (>30 min long) intervals with wind speeds above the thresholds of 50 km/h (approximately intensity 7 in the Beaufort scale, or 14 m/s) and of 65 km/h (18 m/s). These rose diagrams are summarized in Figure 6.

The important outcome of this thresholding is a contrast in the northerly and southerly directions of strong wind between 2007–2009 and 2009–2013. The 2007–2009 interval included the 2008 icing event. The latter was unexpected for the area (minimum repeat interval probably of 60–100 years), included strong northerly winds and caused extensive damage in the infrastructure: exposed water pipes and solar water heater tanks were broken, while their connections with concrete surfaces etc. were damaged because of an ice interface which caused extrusion of clamping bolts. This icing episode accounts for observed screw/bolt extraction in this bridge (Figure 5).

In order to further investigate the contrast in directions of strong winds, the latter were divided into two classes according to their direction relative to the bridge (cf. [17]), northerly and southerly winds (i.e., winds with azimuths 315° < *θ_i_* < 43° and 43° < *θ_i_* < 313° respectively, deck azimuth *φ* = 43°). Furthermore, the component normal to the bridge deck *W_i_*^N^ of northerly winds *W_i_* was computed using the transformation:

(2) $$W_{i}^{N}=\{\begin{array}{ccc} W_{i}(cos\;(\theta_{i}-\varphi ), & \mathrm{if} & 0^{{}^{\circ}}<\theta_{i}<43^{{}^{\circ}}or\;223^{{}^{\circ}}<\theta_{i}<0^{{}^{\circ}}\\ 0, & \mathrm{if} & 43^{{}^{\circ}}<\theta_{i}<223^{{}^{\circ}} \end{array}\;i\;=\;1,\;2,\;\ldots \;n,\;\varphi \;=\;43{}^{\circ}$$

**Figure 8:**
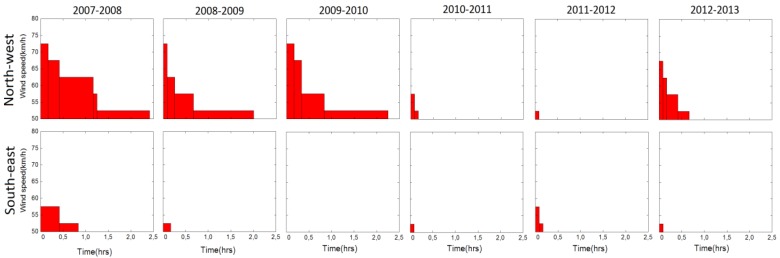
Selective occurrence of strong northerly wind components *W_i_*^N^ during the six intervals defined by the seven measurement surveys. Absence of strong southerly wind components (*W_i_^S^*) justifies the scenario of Figure 10.

A similar transformation was used to compute the corresponding component $W_{i}^{s}$ of winds flowing from a southerly direction. Then, values of $W_{i}^{N}$ and of *W_i_^S^* above the threshold of 50 km/h (which roughly corresponds to the limit between 6 and 7 intensity in the Beaufort scale, ~14 m/s) were selected and are summarized in Table 1 and in Figure 8. These results indicate a characteristic non-uniformity in the distribution of the lateral loading of the bridge by wind during the study interval.

Vortex shading effects may have been important for the Kanellopoulos Bridge, especially because of the transversal wooden beams at its roof (Figure 1 and Figure 5), but are not the focus of this study because no high-rate wind records are available; in addition, vortex shading effects tend to produce quasi-symmetric oscillations and hence cannot explain asymmetric longitudinal splitting cracks.

## 7. Damage Scenario

Previous discussion indicates that damage in the bridge occurred mostly in the interval 2007–2009. This damage is reflected (i) in a dramatic shift of the first lateral modal frequency of the bridge, from 2.6 to 1 Hz (Figure 2), which is much above any statistical error ([24] and Figure A2); (ii) in loss of stiffness of key structural elements, especially of its arch superstructure; (iii) in asymmetric structural damage (longitudinal splitting cracks around bolts in the southerly sides of the bridge).

The interval 2007–2009 is marked by an icing event, as well as by unusual strong northerly wind events (Figure 6 and Figure 8), for which only low-frequency recordings are available. These wind events were characterized by stability in their direction and by amplitude over a certain threshold (Figure 7), and they were most probably combined with gusts, the details of which are not known because of the low frequency of the available wind data, but were also flowing from the same direction with recorded winds (see above).

**Figure 9:**
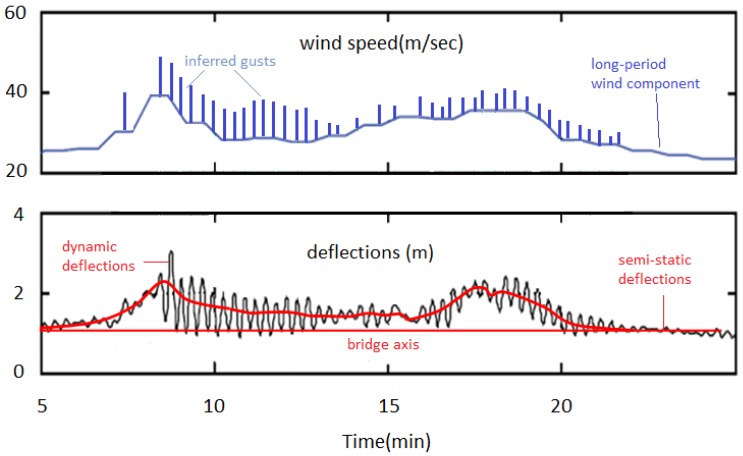
Wind excitation and lateral deflections of the Humber Bridge, UK, based on data of [5]. The time series of deflections imply a combination of semi-static deflections (due to the long-period component of wind), on which are superimposed dynamic deflections (with similar order of magnitude, amplitude up to 2 m) generated by gusts, schematically indicated in the top figure. A similar causative relationship between wind components and deflections is assumed for the Kanellopoulos Bridge as well.

**Figure 10:**
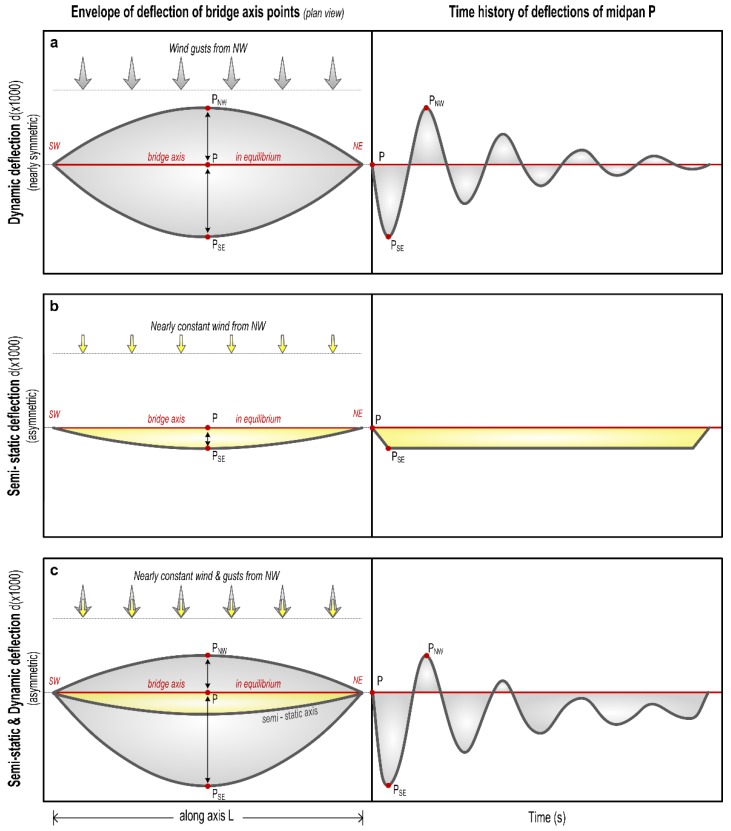
Conceptual model to explain asymmetric bending of the bridge main axis (and hence increased damage), as a result of loading by dominant northerly winds producing a combination of semi-static and dynamic deflections. Inferred from observations in the Humber Bridge (Figure 9) and from results of monitoring surveys. Left column indicates the envelope of all possible positions of the deforming bridge axis, right column the time series of deflections of the middle point. Upper row: Dynamic loading produces essentially symmetric deflections relative to the main bridge axis (when in equilibrium position). Middle row: Semi-static loading produces asymmetric deflections (in yellow). Lower row: The combination of semi-static and dynamic loading (gusts superimposed on nearly steady-state wind load) produces *asymmetric* deflections and strong stresses along the southern sides of the beams. Accumulation of such oscillations produced fatigue-induced cracks (longitudinal splits). This pattern corresponds to the deformation of the midspan of the bridge, 26.5 m long (see Figure 1).

The combination of wind of nearly constant flow with gusts is expected to the have an effect on the Kanellopoulos bridge similar to that recorded in Humber Bridge [5]: semi-static lateral bending of the deck, on which were superimposed dynamic oscillations produced by (inferred) gusts (Figure 9). This leads to an overall asymmetric dynamic loading and deformation pattern, different from that in common symmetric excitations (Figure 3a). The pattern of the response of the Humber Bridge to winds and gusts can be adopted for the Kanellopoulos Bridge, as is analyzed in qualitative form in Figure 10. Because of the asymmetry of this wind-induced dynamic deformation (Figure 8), the southern sides of the deck are subject to cycles of intense bending (extension) producing higher stresses perpendicular to the grain and joints, and leading to micro-cracks/incipient damage; evidence for the latter provide the sounds of creaking (hardly recorded in the video Supplement in [2]). Numerous cycles of this type of loading (though smaller than what is necessary for metallic structures for instance) produce fatigue-generated damage. We may hence correlate damage with excitations and propose the following scenario for the causes of the damage of the timber bridge as a function of time (see Figure 2).

### 7.1. Stage 1, 2000–November 2007

For seven years after its construction, the bridge was stiff (first vertical and lateral modal frequency within specifications, >5.0 and >2.5 Hz, respectively, cf. [4]). In particular, during the first instrumental survey in November 2007 no sign of asymmetric longitudinal splitting cracks etc. existed (Figure 5), and the bridge was very stiff (first lateral modal frequency 2.6 Hz) with the potential of small lateral deflections, as our first field survey indicated.

### 7.2. Stage 2, November 2007–February 2008

Three strong wind events with total duration of 20 h of wind normal to the bridge (recorded mean wind velocities 40.8–51.3 km/h, and maximum recorded velocities between 59.5–72.4 km/h, probably excluding gusts because of low frequency recordings) are expected to have produced numerous cycles of strong lateral excitation and hence fatigue, which is typically localized in areas of stress concentration, usually connections [25]). Such damage is consistent with information for sounds of creaking, and with evidence of weakening of metallic connections (slight extrusion of screws and shearing of the improper x-brace stiffeners at the superstructure; Figure 5). As a result, the rigidity of the arch superstructure was reduced.

### 7.3. Stage 3, 17–19 February 2009 Flash Ice Event

This extraordinary for the area icing event (temperature 0 to −1.8 °C for about 18 h) followed a rain interval during which water was sandwiched between connections wood-to-wood and wood-to-metal (screws etc.). Because of the transformation of water to ice, widespread damage was caused to many structures in the area (for example broken water pipes, solar panels detached from their bases, etc.). In the study bridge, this icing event is inferred to have damaging effects as well: because of poor craftsmanship (imperfect clamping of metal elements to wood, etc.) and because some connections were already loosen since Stage 2, transformation of the water interface into ice caused extrusion of some screws (Figure 5) and rendered nearly useless the already weakened stiffeners at the bridge roof (transversal beams at the upper parts of the two arches and X-braces, Figure 5). The overall effect was to highly reduce the lateral stiffness of the bridge.

### 7.4. Stage 4, February 2008–May 2009

Strong northerly winds produced numerous cycles of asymmetric excitation of the bridge, as explained in Figure 9 and Figure 10. Oscillation of the weakened arch superstructure imposed additional load to deck, with higher deflections to its south side. Evidence of this last effect is provided by the difference in the amplitude of oscillations between arch over-structure and deck: For example, during the 2013 survey, during free attenuating oscillations the amplitude of oscillations of the superstructure was 7% higher than that of the deck, and 41% for steady/transient oscillations (see Section 4 above), indicating additional strain imposed on the deck by the oscillating superstructure (see also video in [2]). Numerous cycles of this deformation are expected to have amplified longitudinal splitting cracks near connections. Shaking by a magnitude 6.4 earthquake in June 2008 may have contributed in the bridge decay. As a result of these effects, in May 2009 the first lateral frequency of the bridge was reduced to 1.0 Hz.

### 7.5. Stage 5, May 2009–May 2013

Additional strong northerly wind episodes amplified damage. Because of the reduction of its lateral stiffness, the bridge had become vulnerable to weaker winds and to human-induced excitations; in fact it became popular to groups of youngsters who produced lateral, though symmetric excitations. This is reflected in a slightly decreased main lateral frequency (Figure 2).

### 7.6. Stage 6, May 2013–Till 2017

Preliminary instrumental data indicate slight intensification of damage and additional slight reduction of the main lateral modal frequency. However, especially after 2015, increase of longitudinal splitting cracks in most parts of the bridge were observed, while non-bearing elements show signs of intense decay.

## 8. Discussion

Previous analysis suggests that damage of the bridge, and especially the asymmetric longitudinal splitting cracks, are due to effects more complicated than what was initially assumed [2]. Evidence presented above indicates that taking advantage of constructional vicissitudes (lack or improper stiffeners, Figure 1c and Figure 5b, etc.), the combination of an icing episode, for which the bridge was unprepared, and of strong winds selectively flowing from the north (Figure 8) produced asymmetric oscillations (Figure 10) amplified by the load imposed by the weakened superstructure (Figure 1d). This is a realistic scenario because the length of the midspan is large enough to permit bending (26.5 m; Figure 1), and no significant signs of damage were observed at the connections of the horizontal beams with the four vertical columns. Loading of the deck by the two arches produced minor torsion only, as was derived from monitoring data indicating no significant elevation differences at both sides of the deck during oscillations. This is mostly due to the high stiffness of the bridge along the vertical axis (see above).

During our field surveys and during oscillations induced by strong winds, sounds of creaking were recorded. The latter probably reflected crack initiation and then transition to the crack propagation stage (cf. [26]; for an analogy with steel bridges, see [27]). This reflects the crucial role of the tensile stress perpendicular to the grain on the joints, and this behavior reflects a particularity of timber structures [9].

Fatigue is assumed to have played a major role in the observed damage, especially the asymmetric slits around connections. This is because in timber structures high strain and damage are indeed concentrated around connections [25]. Longitudinal splitting cracks along connections may have been amplified by racking (cf. [28]), i.e., exceedance of the capacity of wood to shear deformation at the screws due to high displacement imposed by the extreme deflections, especially since damage stage 4.

Our preferred scenario is that initial damage by the icing event was amplified due to tensile stress produced by numerous strong wind events. The latter were characterized by a rather constant wind combined by gusts, both from a nearly stable direction (Figure 7), causing a semi-static lateral bending on which were superimposed high-frequency dynamic oscillations of the same order of amplitude, in analogy to Figure 9, as explained in Figure 10. Wind excitation was direct on the deck, and indirect, from the increased lateral swaying of the arch superstructure, and produced stresses which in timber structures tend to be concentrated in connections, and to produce strain normal to grain [25]. Both effects produced asymmetric strain, which explains splitting along the southern joints between deck and arch. Each cycle of strong northerly wind loading tended to produce (or amplify) an infinitesimal crack, as sound of creaking indicates.

A main problem is how to document the necessary number of cycles of asymmetric dynamic deformation to explain observed splitting. Wind gusts, evidence of which may provide some peaks in Figure 7, typically last for up to 20 s [29], and they occur every few minutes during high wind intervals, as observations in the wider region indicate. Based on data of Table 1, hundreds of cycles of gust loading between 2007 and 2010 are inferred. Clearly, such amount of cycles of deformation is small to produce fatigue-induced damage in metallic structures, but it is clearly enough for timber structures, especially under high strain rates [25], i.e., extreme lateral deflections. Fatigue can be claimed also responsible for longitudinal splitting cracks with symmetric distribution, resulting from dynamic oscillations of various types, including Karman-vortex effects and crowd-loading effects, some intentional.

Another point discussed in this article is that superimposition of transient, dynamic wind effects on semi-static (quasi-steady state) wind effects is a problem known since long [30], but its significance was recently only realized even for tall buildings [31]. In this study, impacts of such superimposition are examined for a small structure.

**Figure A1:**
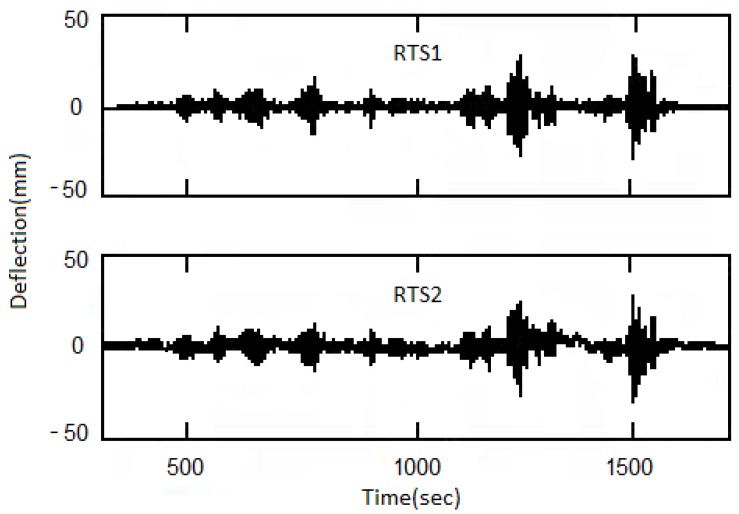
Lateral deflections of the bridge during certain excitation events of the 2009 survey recorded by two different robotic theodolites (RTS) sighting at reflectors at the two sides of the bridge, at its midspan. Amplitude of deflections is similar for the two instruments, with a maximum of the order of ~25 mm. Recorded apparent deflections during the intervals of no excitation define the static noise level of the RTS, 1–2 mm [35].

**Figure A2:**
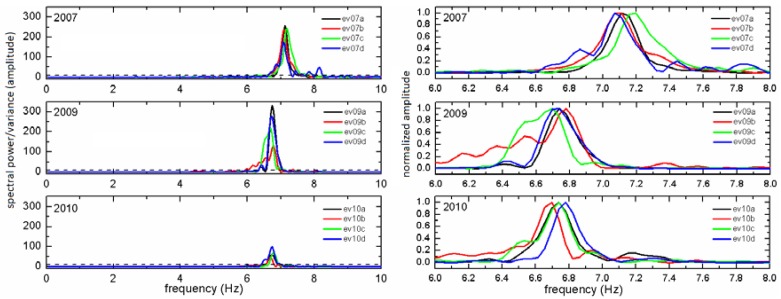
Superimposed accelerometer-derived spectra of selected free attenuating vertical oscillations during the 2007, 2009 and 2010 surveys, normalized to their maximum value (**left**) and zoomed around the dominant peak (natural frequency, **right**). Mark consistency of curves, permitting to define vertical modal frequencies of the bridge with resolution of a fraction of a Hz. The shift between 2007–2009 and the absence of shift between 2009–2010 is consistent with damage derived from lateral deflections, and indicates no significant structural damage along the vertical axis (after [24]).

This study focusses on lateral deflections. Hence a possible question is whether previous results can be modified if vertical deflections and variations of the first vertical mode are examined. In fact, evidence presented by [7,32] and summarized in Figure A1 indicates that in the interval 2007–2010 (stages 1 to part of stage 5) only a slight change in the vertical modal frequency of the bridge was documented, probably related to the icing episode, and the bridge has remained stiff along the vertical axis (modal frequency ~6.5 Hz), and no major deformation associated with vertical deflections (including torsion of the deck because of the swaying arch superstructure) is expected.

## 9. Conclusions

Decay of timber structures is a rather slow process, mostly due to environmental effects, and usually related to reduction of their moisture content, which in turn reduces their strength or induces stresses (for example [33,34]); for this reason, many, especially old timber bridges, are roofed for protection.

The Kanellopoulos bridge represents an exceptional case of timber bridge damage because its structural evolution was very rapid (in only a few years) and was to a major extent due to dynamic effects. Apart from an initial reduction of its stiffness because an unexpected environmental effect (icing episode) which played a catalytic role because of structural and constructional vicissitudes (omission of X-bracing beneath the deck, improper connections permitting an ice interface in an area free of ice), the structural decay was due to wind loading producing asymmetric semi-static and dynamic deflections of high amplitude, of the order of >2–10 cm, unusual for common and much longer timber bridges (cf. [17]). This was possible because fatigue appears in wood after fewer cycles of strain than in other structures, and wood is vulnerable to tensile stress normal to the grain especially at connections. An important aspect of this study is that it is not simply based on analysis of experimental data to detect damage [6], but on a combination of analysis of monitoring and of wind data with observations of damage and with evidence from other bridges.

## Figures and Tables

**Table 1:** Frequency and total duration of wind events/subevents with component >50 km/h normal to the bridge, flowing from northerly (WiN) and southerly (WiS) directions (see also Figure 8). The icing event of 2008 and damage effects are also summarized.

Interval	*W_i_*^N^ > 50 km/h		*W_i_^S^* > 50 km/h		Effects
	Events & Subevents	Cumulative Duration (min)	Events & Subevents	Cumulative Duration (min)	
September 2007–February 2008	14	70	5	25	F = 2.6 to 1.1 Hz; superstructure damage; asymmetric longitudinal splitting cracks
Icing event (17–19 February 2008)					
February 2008–May 2008	15	75	5	25	
May 2008–May 2009	24	120	2	10	
May 2009–May 2010	27	135	0	0	F = 1.1 to 0.95 Hz; asymmetric longitudinal splitting cracks amplified
May 2010–May 2011	2	10	1	5	
May 2011–May 2012	1	5	2	10	
May 2012–May 2013	8	40	1	5	

## Appendix A. Bridge Monitoring: Instrumentation, Techniques and Results

### Appendix A.1. Overview

The analysis of the dynamic characteristics of the Kanellopoulos bridge during yearly surveys was based on controlled forced excitations of the bridge by coordinated motion (jumps etc.) of a group of pedestrians (total mass of >500 kg). The aim was to excite the bridge (produce transient oscillation) until the bridge deflections were important, giving the impression of impeding collapse. Then the group instantly froze, producing a free attenuating oscillation, dominated by its first modal frequency (lateral or vertical), as is shown in Figure 3. A video with a characteristic oscillation is available in the Supplement of [2].

Each yearly survey lasted for hours, contained numerous excitations under similar environmental conditions, and several valid excitations were selected based on accelerometer recordings (see Figure 3 and Figure A1). Although excitation conditions were very similar, in some surveys participating students were more daring, leading to larger deflections, but modal frequencies during each survey were slightly only influenced (Figure A2, [32]). In addition, intervals of movement and of no movement alternated, so that the equilibrium conditions (ambient noise in measurements, etc.) and the noise threshold of sensors can be determined.

### Appendix A.2. Instrumentation

Bridge excitation was recorded by several sensors: accelerometers, geodetic sensors and cameras (video-recording (see for example Supplement in [2] and photos). Numerous sensors or sensor parts were rigidly clamped on selected points of the bridge so that its full structural response is accurately controlled; emphasis was given to collocated sensors. However, measurements focused on the middle span of the deck, in the two sides of which an accelerometer, a reflector of robotic theodolite (RTS) and a GPS receiver were fixed on top of each other. Synchronization was made either directly through GPS timing (GPS, accelerometers) or through statistical analysis of selected movements of RTS reflectors collocated with GPS receivers.

Geodetic sensors: Two RTS were placed on stable ground, about 70–100 m from the bridge and each was tracking a high accuracy prismatic reflector clamped on the deck. The instantaneous polar coordinates of the reflectors were measured with an average sampling frequency of 7 Hz and uncertainty threshold of up to 2 mm (Figure A1). In some surveys, a MA 100 distance measurement tellurometer with a dedicated reflector was used. Its accuracy is 0.5 mm, and its sampling rate 60 Hz. Figure 3a shows the nearly perfect recording of a representative oscillation. Details in [2,7,24,32,35].

Several GPS receivers with a sampling rate of 10 to 100 Hz were fixed on parts of the bridge and on stable ground (reference sensors for “differential” positioning). Their accuracy in coordinate changes after filtering and weak fusion with accelerometers is of the order of 1 to a few mm.

Accelerometers: Two typical forced balance accelerometers (sampling frequency 250 Hz) with GPS timing and in some surveys, additional high-accuracy MEMS accelerometers were used (for details see [24]).

### Appendix A.3. Data Analysis and Results

Recordings of all sensors were analyzed with techniques fully explained in [2,24,35] and their final output was mostly 3-D deflections and accelerations during excitations and the intervals between them. Deflections in the longitudinal axis of the bridge were insignificant, along the vertical were up to 3 mm, while lateral deflections were ranging to about 20 mm (2007 and 2009 surveys) and over 60 mm (later surveys). These deflections are absolutely significant from the statistical point of view (compared to the standard error of instruments) and from structural/logical constraints (comparison of recordings of intervals of no motion and of excitation, cf. Figure A1).

Timeseries of free attenuating oscillations were then tested whether they follow the typical model of Equation (3) [16]:

(3) $$f\left(x\right)=e^{-\omega \xi t}\sin(\omega t+\varphi )$$

(where *ω* = 2π*f* constant angular velocity, *f* modal frequency, *ξ*: damping factor, *φ*: phase and *t*: time), i.e., whether they correspond to a SDOF and hence describe a first modal frequency. A characteristic result is shown in Figure 3b.

From the tested time series of deflections and of accelerations, spectra were computed. The frequency resolution of RTS-derived spectra is 0.007 Hz, and 0.004 Hz for the GNSS- and accelerometer-derived spectra. However, because of various limitations (short attenuation intervals, slight variations of loading conditions in each excitation, peak spectral values differing by usually 0.01–0.02 Hz were found. This is exemplified in Figure A2, showing accelerometer spectra for different vertical oscillations at the midspan of the bridge for selected surveys (events, curves marked with a different colour).
